# The relationship between serum HDL-cholesterol, cardiovascular disease and mortality in community-based people with type 2 diabetes: the Fremantle Diabetes Study phase 2

**DOI:** 10.1186/s12933-024-02447-0

**Published:** 2024-10-14

**Authors:** Timothy M. E. Davis, S. A. Paul Chubb, Wendy A. Davis

**Affiliations:** 1 Medical School, University of Western Australia, Fremantle Hospital, PO Box 480, Fremantle, WA 6959 Australia; 2 Department of Endocrinology and Diabetes, Fiona Stanley and Fremantle Hospitals, Murdoch, WA Australia

**Keywords:** Type 2 diabetes, HDL-cholesterol, Major adverse cardiovascular events, Mortality, Sex differences

## Abstract

**Background:**

Older general population-based studies found an inverse association between serum HDL-cholesterol and both cardiovascular disease (CVD) events and mortality, but more recent data have suggested a U-shaped relationship. Whether this applies to type 2 diabetes is uncertain. The aim of this study was to assess the prognostic significance of serum HDL-cholesterol concentrations in representative, community-based participants from the Fremantle Diabetes Study Phase II (FDS2).

**Methods:**

We followed 1,479 FDS2 participants with confirmed type 2 diabetes (713 females, mean age 65.6 years; 763 males, mean age 65.9 years) from entry (2008–2011) to death/end-2021. Major adverse cardiovascular events (non-fatal myocardial infarction (MI), non-fatal stroke, cardiovascular death; 3-point MACE), and all-cause mortality were ascertained from prospectively collected data and validated administrative databases. Independent associates of 3-point MACE by sex, excluding participants with prior MI/stroke, were assessed using Cox and competing risk models with sex-specific quintiles of HDL-cholesterol added to the most parsimonious models. Predictors of all-cause mortality were identified using Cox proportional hazards modelling.

**Results:**

In females, with baseline serum HDL-cholesterol quintile 2 (1.04–1.22 mmol/L) as reference, both quintiles 1 (< 1.04 mmol/L) and 5 (> 1.59 mmol/L) were significant independent predictors of 3-point MACE (*P* < 0.027) and all-cause death (*P* < 0.019) after adjustment for a full range of demographic, clinical and laboratory variables. In males, serum HDL-cholesterol quintile did not add to the most parsimonious model for 3-point MACE, but quintile 1 (< 0.90 mmol/L) was a significant predictor of death (*P* = 0.026 versus quintile 4 (1.15–1.31 mmol/L) as reference) after adjustment. Competing risk analyses for 3-point MACE showed similar results to the Cox models for both sexes.

**Conclusion:**

There was a significant U-shaped relationship between serum HDL-cholesterol and both 3-point MACE and all-cause death in females with type 2 diabetes after adjustment for confounders. There was no such relationship for 3-point MACE in males but a low HDL-cholesterol was associated with all-cause mortality. These data have sex-specific implications for assessment of serum lipid profiles in the clinical management of type 2 diabetes.

## Background

Although older general population epidemiological studies found a simple inverse association between serum HDL-cholesterol concentrations and both cardiovascular disease (CVD) events and mortality [1, 2], a range of data over the last decade have suggested that there is a more complex U-shaped relationship [3–8]. Whether this applies in the specific case of type 2 diabetes is uncertain. Although there are quantitative, qualitative and functional changes in HDL-cholesterol associated with diabetes which may be important in determining a distinctive role in cardiovascular pathophysiology [9], available prospective data from studies involving people with diabetes are inconsistent.

The first report of a non-linear association in diabetes came from the Pittsburgh Epidemiology of Diabetes Complications study in which there was a U-shaped association for women, and a linear inverse association for men, with type 1 diabetes and the risk of coronary artery disease events [10]. More recent analyses of large-scale administrative data from people with diabetes of unspecified type from the US [11], Japan [12] and China [13] have demonstrated (i) an increase in CVD events that was restricted to only relatively high serum HDL-cholesterol concentrations after adjustment for confounders [13], (ii) a U-shaped relationship for the composite of myocardial infarction (MI), stroke and all-cause death that was greater for men than women in unadjusted analyses [12], and (iii) a U-shaped relationship for all-cause and cardiovascular disease mortality in adjusted analyses [11]. Differences in sample characteristics, use of sex-specific analyses, and the range of available confounding variables all complicate comparisons between these studies.

Since regular measurement of HDL-cholesterol as a component of a serum lipid profile is recommended as part of routine diabetes care [14], and because there are sex differences in its value in predicting cardiovascular risk [15], there is a need for an assessment of the relationship between serum HDL-cholesterol and both CVD events and mortality in contemporary unselected cohorts of males and females with well-characterised type 2 diabetes. The aim of this study was, therefore, to assess the prognostic significance of serum HDL-cholesterol concentrations in representative, community-based participants from the Fremantle Diabetes Study Phase II (FDS2).

## Methods

### Study site, participants and approvals

The FDS2 is a longitudinal, observational study of diabetes conducted in a postcode-defined geographical area based around the port Fremantle in Western Australia (WA). Eligible participants were identified from hospital inpatient and outpatient databases, primary care and specialist practices, pharmacies, optometrists and opticians, third-party mail-outs to registrants of the National Diabetes Services Scheme and the National Diabetes Register, local advertising, and word of mouth [16]. Details of recruitment strategies, the FDS2 sample (mean age 62 years, 52% males and 90% with clinically defined type 2 diabetes), and eligible but non-recruited people (mean age 61 years, 52% males and 90% with clinically defined type 2 diabetes) have been published [16]. Data relating to income, employment, housing, transportation and other socio-economic variables in the study catchment area have been used to calculate an average Index of Relative Socio-economic Advantage and Disadvantage of 1033 with a range by FDS2 postcode of 977 to 1,113, figures similar to the Australian national mean ± SD of 1,000 ± 100 [17]. The FDS2 protocol was approved by the Human Research Ethics Committee of the Southern Metropolitan Area Health Service (reference 07/397). All participants gave written informed consent. No clinical trial registration was required.

**Fig. 1:**
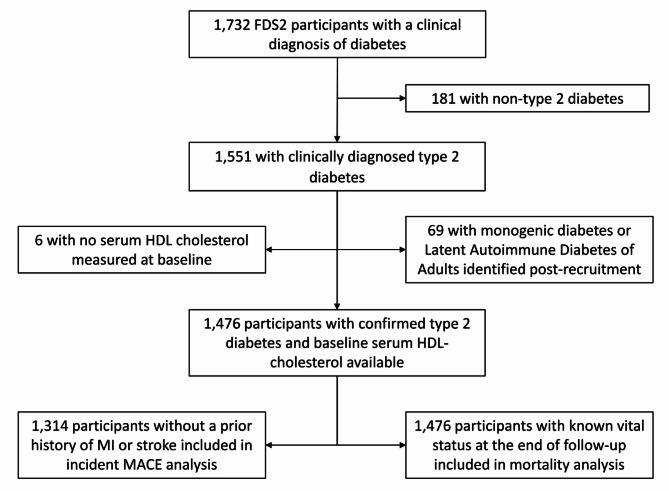
Consort diagram showing the participants in the Fremantle Diabetes Study Phase II included in the present study

Of 4,639 people with known clinician-diagnosed, non-gestational diabetes found between 2008 and 2011 in a population base of 150,000, 1,668 (36.0%) were recruited. Sixty-four participants from the first phase of the FDS recruited between 1993 and 1996 from the same catchment area as FDS2 but who were resident elsewhere at the time of FDS2 recruitment were also enrolled (total cohort 1,732, of whom 1,551 had type 2 diabetes). For the purposes of the present study, there were 1,476 participants with confirmed type 2 diabetes after those with monogenic forms and Latent Autoimmune Diabetes of Adults had been excluded [18], and whose serum HDL-cholesterol was measured at baseline (see Fig. 1).

### Clinical and laboratory assessments

All FDS2 participants were invited to face-to-face assessments at entry and then biennially over the next six years [16]. At each assessment, a standardised comprehensive questionnaire was completed and a physical examination was performed, and blood and urine samples were sent for fasting biochemical tests performed in a single nationally accredited laboratory. Participants were requested to bring details of all medications to each visit. Racial/ethnic background was categorised according to self-selection, country/countries of birth and parents’/grandparents’ birth, and language(s) spoken at home as either Anglo-Celt, Southern European, Other European, Asian, Aboriginal or mixed/other. Body mass index (BMI) was calculated, together with a body shape index (ABSI) which represents a more reliable estimate of visceral adiposity in relation to mortality [19]. Chronic diabetes complications were identified using standard definitions [16]. Urinary albumin: creatinine ratio (uACR) was determined from a first-morning sample and renal impairment from the estimated glomerular filtration rate (eGFR) [20]. Distal symmetrical polyneuropathy (DSPN) was defined using the vibration perception threshold [21]. Peripheral arterial disease (PAD) was defined as an ankle brachial index ≤ 0.90 or a diabetes-related lower extremity amputation.

Serum HDL-cholesterol was determined by homogenous enzymatic methods, initially using reagents and an Integra 800 analyser supplied by Roche Diagnostics, NSW, Australia, and subsequently using reagents and an Architect ci8200 analyser supplied by Abbott Diagnostics, NSW, Australia. Results by the Roche method were adjusted to Abbott-equivalent values to ensure comparability across the study [22]. Assay imprecision was 5.3% at 0.6 mmol/L and 4.0% at 2.1 mmol/L. In addition to standard care assays, serum N-terminal pro-brain natriuretic peptide (NT-proBNP) was measured on an Elecsys 2010 (Roche Diagnostics Australia) and serum C-reactive protein was measured on an Architect ci16200 analyser (Abbott Diagnostics Australia, North Ryde, NSW, Australia) using a high-sensitivity protocol (hsCRP) with reagents supplied by Abbott Diagnostics. In view of the fact that metabolic dysfunction-associated fatty liver disease (MAFLD) is relatively common in type 2 diabetes, especially amongst women [23], and that it can attenuate serum HDL-cholesterol concentrations [24], we also measured analytes (in addition to conventional liver function tests) that have associated with its presence [25], specifically serum apolipoprotein A1, serum hyaluronic acid, serum haptoglobin and serum alpha-2 macroglobulin. Serum apolipoprotein A1 and haptoglobin were measured by immunoturbidimetry on an Architect 16,200 analyser (Abbott Diagnostics) using reagents supplied by Abbott Diagnostics. Serum alpha-2 macroglobulin and hyaluronic acid were measured on a Cobas 501 (Roche Diagnostics) analyser using reagents supplied by Roche Diagnostics.

### Ascertainment of cardiovascular outcomes and deaths

The Hospital Morbidity Data Collection (HMDC) contains validated data relating to all public and private hospitalisations in WA from 1970 onwards, and the Death Register contains information on all deaths in WA starting from 1969 [26]. The FDS2 database has been linked to these data sources through the WA Data Linkage System (WADLS) to end-December 2021, as approved by the WA Department of Health Human Research Ethics Committee. Data from the HMDC was used to supplement information obtained at FDS2 assessments relating to prior or prevalent complications or co-morbidities reported during the five years prior to entry. A history of ischaemic heart disease (IHD) or cerebrovascular disease were defined as hospitalisations or death with/for/of IHD or cerebrovascular disease, respectively, before FDS2 recruitment. Incident major adverse cardiovascular events (MACE) were defined as hospitalisations or deaths with/for/of MI, stroke or cardiac/cerebrovascular/sudden death. HMDC data were used to calculate the Charlson Comorbidity Index (CCI) [27] excluding conditions that were coded as diabetes-specific. Causes of death on the death certificate or obtained from a coroner’s report were reviewed independently by two FDS2 physicians and classified under the UK Prospective Diabetes Study coding system [28]; in the case of discrepancy, the casenotes were consulted and a consensus obtained.

#### Statistical analysis

The computer packages IBM SPSS Statistics 29 (IBM Corporation, Armonk, NY, USA) and StataSE 15 (College Station, TX: StataCorp LP) were used for statistical analysis. Data are reported as percentage, mean ± SD, geometric mean (SD range), or, when variables are not approximately normally distributed, median [interquartile range]. Two-way comparisons were performed using Fisher’s exact test for independent samples, Student’s *t*-test for approximately normally distributed variables, and Mann-Whitney U-test for non-normally distributed variables. Serum HDL-cholesterol was examined both as a continuous variable and as sex-specific quintiles.

Cox proportional hazards modelling (backward conditional variable selection with *P* < 0.050 for entry and ≥ 0.050 for removal) was used to identify independent baseline predictors of incident MACE and all-cause mortality by sex. All clinically plausible variables (excluding serum HDL-cholesterol) with bivariable *P* ≤ 0.20 were considered for model entry in a backward stepwise manner. These included demographic and diabetes-related factors, the presence of non-MACE complications, cardiovascular risk factors and markers of liver disease risk. Sex-specific quintiles of serum HDL-cholesterol were added to the most parsimonious models excluding serum HDL-cholesterol as a continuous variable. The proportional hazards assumption was assessed and, if violated, significant time-varying covariates were included in Cox models. A two-tailed significance level of *P* < 0.05 was used throughout. Fine and Gray competing risk regression modelling was also performed in the case of incident MACE to adjust for the competing risk of death from causes other than CVD [29]. Restricted cubic spline modelling with three, four and five knots was undertaken to confirm the shape of the relationship between HDL-cholesterol and incident MACE and all-cause mortality by sex.

## Results

### Baseline participant characteristics

We followed 1,476 FDS2 participants with confirmed type 2 diabetes. Of these, 713 were females of mean ± SD age 65.6 ± 11.9 years and they had a median [IQR] diabetes duration of 8.0 [3.0–16.0] years. The 763 males had a mean ± SD age of 65.9 ± 11.2 years and their diabetes duration was 10.0 [2.9–15.9] years. At study entry, 24% of our cohort was on diet-based glycaemic management, just over two-thirds were taking oral medications, and 22% were insulin-treated. Metformin (64%) and sulfonylureas (31%) were the most common medications, with much lower use of thiazolidinediones (6%), dipeptidyl peptidase 4 inhibitors (2%), acarbose (1%), exenatide (0.1%) and repaglinide (0.1%). None was taking a sodium-glucose cotransporter 2 inhibitor.

### Incident major adverse cardiovascular events

There were 665 females and 649 males without a prior history of MI or stroke (93.2% and 85.1%, respectively, of FDS2 participants by sex at baseline; see Fig. 1) who were followed until they experienced a first MACE or died from other causes or end-2021, whichever came first.

In the females, 144 (21.7%) had a MACE during 10.3 ± 3.5 (range 0 to 13.8) years of follow-up. Compared to those who did not have a MACE, bivariable analyses (see Table 1) showed that those who did were older, more likely Indigenous Australian and less likely Asian, were less likely to be married/in a *de facto* relationship, were less likely to have been educated beyond primary level, had longer duration diabetes with worse glycaemic control despite a greater likelihood of insulin therapy, were more likely to be obese by ABSI but not BMI, had higher systolic blood pressure and total serum cholesterol, were more likely to have had prior CVD (excluding MI and stroke) and were more likely to be taking aspirin, and had a higher likelihood of anaemia, retinopathy, PAD, DSPN and nephropathy (lower eGFR and greater uACR). In relation to non-standard-of-care biomarkers and indices of liver dysfunction, those with an incident MACE had higher serum NT-proBNP, serum aspartate/alanine transferase ratio (AST/ALT), serum hyaluronic acid (ug/L) and serum alpha-2 macroglobulin (g/L).

**Table 1:** Baseline characteristics by three-point MACE to end-2021 by sex in people with type 2 diabetes excluding those with a prior history of MI or stroke

	Females			Males		
	No 3-point MACE	3-point MACE	*P-*value	No 3-point MACE	3-point MACE	*P-*value
Number (%)	521 (78.3)	144 (21.7)		490 (75.5)	159 (24.5)	
Age (years)	63.8 ± 11.3	69.6 ± 12.4	< 0.001	63.7 ± 11.3	68.7 ± 9.8	< 0.001
Ethnic background (%):			0.002			0.518
Anglo-Celt	55.3	52.1		52.7	47.2	
Southern European	10.7	13.2		14.9	13.2	
Other European	6.5	4.9		7.8	10.1	
Asian	5.4	0.7		4.5	7.5	
Aboriginal	6.9	16.7		4.3	4.4	
Mixed/other	15.2	12.5		15.9	17.6	
Not fluent in English (%)	9.8	13.2	0.282	11.2	13.2	0.481
Currently married/*de facto* relationship (%)	54.9	44.4	0.030	71.8	74.2	0.610
Educated beyond primary level (%)	87.9	80.9	0.047	88.2	85.9	0.484
Smoking status (%)			0.209			0.278
Never	58.5	51.7		30.6	27.8	
Ex	33.0	35.7		58.3	56.3	
Current	8.5	12.6		11.1	15.8	
Alcohol consumption (standard drinks^a^/day)	0.1 [0-0.3]	0 [0-0.6]	0.314	0.8 [0.1–1.8]	0.3 [0-1.5]	0.009
Age at diabetes diagnosis (years)	55.0 ± 11.9	56.5 ± 15.1	0.294	54.5 ± 11.5	56.3 ± 10.9	0.077
Diabetes duration (years)	7.0 [2.0–15.0]	12.0 [6.0-18.2]	< 0.001	7.4 [2.0–15.0]	12.0 [5.0-17.5]	< 0.001
Diabetes treatment (%)			0.006			0.026
Diet-based	30.9	21.7		21.6	12.6	
Oral glucose lowering agents (OHAs)/non-insulin injectables	51.4	49.0		58.2	59.1	
Insulin only	3.8	9.1		4.7	7.5	
Insulin ± OHAs/non-insulin injectables	13.8	20.3		15.5	20.8	
Fasting serum glucose (mmol/L)	7.1 [6.1–8.5]	7.0 [6.0-9.6]	0.448	7.2 [6.2-9.0]	7.5 [6.2–9.3]	0.560
HbA_1c_ (%)	6.8 [6.2–7.6]	7.0 [6.3-8.0]	0.024	6.8 [6.2–7.7]	7.0 [6.2-8.0]	0.166
HbA_1c_ (mmol/mol)	51 [44–60]	53 [45–64]	0.024	51 [44–61]	53 [44–64]	0.166
BMI (kg/m^2^)	31.9 ± 6.6	31.3 ± 7.0	0.313	31.0 ± 5.8	30.3 ± 4.7	0.117
ABSI (m^11/6^ kg^− 2/3^)	0.080 ± 0.006	0.081 ± 0.006	0.002	0.082 ± 0.004	0.083 ± 0.005	0.249
Central obesity (by waist circumference^b^; %)	80.9	76.9	0.289	63.5	65.2	0.775
Systolic blood pressure (mmHg)	141 ± 21	150 ± 27	< 0.001	146 ± 19	154 ± 22	< 0.001
Diastolic blood pressure (mmHg)	78 ± 12	77 ± 13	0.757	83 ± 11	83 ± 11	0.697
Heart rate (beats/min)	71 ± 12	71 ± 14	0.793	69 ± 12	70 ± 14	0.174
Antihypertensive therapy (%)	69.9	76.8	0.117	70.4	76.7	0.129
Total serum cholesterol (mmol/L)	4.6 ± 1.1	4.8 ± 1.6	0.048	4.2 ± 1.0	4.2 ± 1.1	0.800
Serum non-HDL-cholesterol (mmol/L)	3.2 ± 1.1	3.5 ± 1.6	0.095	3.1 ± 1.0	3.0 ± 1.0	0.724
Serum HDL-cholesterol (mmol/L)	1.32 ± 0.33	1.37 ± 0.39	0.179	1.14 ± 0.30	1.14 ± 0.30	0.756
Serum HDL-cholesterol quintiles (%):			0.139			0.471
1	20.3	22.9		19.4	23.9	
2	24.8	16.7		21.0	16.4	
3	16.9	15.3		20.4	18.9	
4	21.5	21.5		20.6	18.9	
5	16.5	23.6		18.6	22.1	
Serum triglycerides (mmol/L)	1.5 (0.9–2.4)	1.6 (1.0-2.8)	0.050	1.6 (0.9–2.7)	1.6 (1.0-2.6)	0.761
Lipid-lowering therapy (%)	65.9	66.9	0.842	66.5	72.3	0.204
Statin use (%)	63.4	66.9	0.490	64.7	69.2	0.336
Aspirin (%)	31.0	40.1	0.044	34.6	41.8	0.106
Cerebrovascular disease (%)	3.1	9.0	0.004	4.3	8.8	0.041
Coronary heart disease (%)	17.9	34.7	< 0.001	18.6	35.2	< 0.001
PAD (%)	22.9	39.6	< 0.001	14.3	22.8	0.018
DSPN (%)	26.2	39.4	0.003	43.1	48.1	0.311
eGFR category (%)			< 0.001			0.039
≥ 90 mL/min/1.73m^2^	44.8	27.3		41.9	33.5	
60–89 mL/min/1.73m^2^	43.4	44.8		45.4	44.9	
45–59 mL/min/1.73m^2^	6.0	15.4		7.2	12.0	
< 45 mL/min/1.73m^2^	5.8	12.6		5.5	9.5	
uACR (mg/mmol)	2.7 (0.9–8.1)	5.4 (1.2–23.5)	< 0.001	2.8 (0.8–9.6)	4.9 (0.9–26.8)	< 0.001
Any retinopathy (%)	28.0	47.5	< 0.001	37.7	50.0	0.009
Anaemia^c^ (%)	8.4	16.0	0.012	7.8	14.5	0.018
Platelets (x10^9^/L)	267 (208–343)	267 (204–349)	0.971	229 (173–304)	234 (175–311)	0.493
Serum NT-proBNP (pg/mL)	66 (20–218)	166 (43–641)	< 0.001	52 (12–213)	100 (20–496)	< 0.001
Serum hsCRP (mg/L)	3.0 (0.9–9.4)	3.4 (1.1–10.5)	0.199	2.1 (0.7–6.2)	2.1 (0.7–6.2)	0.980
Serum albumin (g/L)	44 ± 3	43 ± 4	0.399	44 ± 3	44 ± 3	0.683
Serum AST/ALT	1.2 (0.8–1.6)	1.3 (0.9–1.8)	< 0.001	1.0 (0.7–1.5)	1.2 (0.8–1.8)	0.003
Serum gamma-glutamyl transferase (U/L)	28 (14–53)	30 (14–66)	0.173	35 (17–72)	33 (16–68)	0.318
Serum bilirubin (µmol/L)	8.9 (5.9–13.5)	8.2 (5.5–12.4)	0.041	10.4 (6.9–15.5)	10.5 (7.1–15.4)	0.787
Serum apolipoprotein A1 (g/L)	1.55 ± 0.25	1.57 ± 0.30	0.368	1.42 ± 0.25	1.41 ± 0.24	0.959
Serum hyaluronic acid (ug/L)	47 (23–98)	62 (31–124)	< 0.001	54 (26–114)	63 (30–132)	0.035
Serum haptoglobin (g/L)	1.64 ± 0.57	1.70 ± 0.57	0.231	1.45 ± 0.59	1.55 ± 0.53	0.067
Serum alpha-2 macroglobulin (g/L)	1.99 (1.45–2.74)	2.34 (1.71–3.20)	< 0.001	1.99 (1.37–2.87)	2.27 (1.63–3.16)	< 0.001

^a^1 standard drink = 10 U ethanol; ^b^≥102 cm in males and ≥ 88 cm in females; ^c^haemoglobin ≤ 130 g/L males, ≤ 120 g/L females

Amongst the males, 159 (24.5%) had a MACE during 9.7 ± 3.7 (range 0.1 to 13.8) years of follow-up. Compared to those who did not have a MACE, bivariable analyses (see Table 1) showed that those who did were older, had a lower alcohol consumption, were more likely to be insulin-treated, had higher systolic blood pressure, were more likely to have had prior CVD (excluding MI and stroke), and had a higher likelihood of anaemia, retinopathy and PAD, and a greater uACR. In relation to non-standard-of-care biomarkers and indices of liver dysfunction, those with an incident MACE had higher serum NT-proBNP, serum AST/ALT, serum hyaluronic acid and serum alpha-2 macroglobulin.

**Fig. 2:**
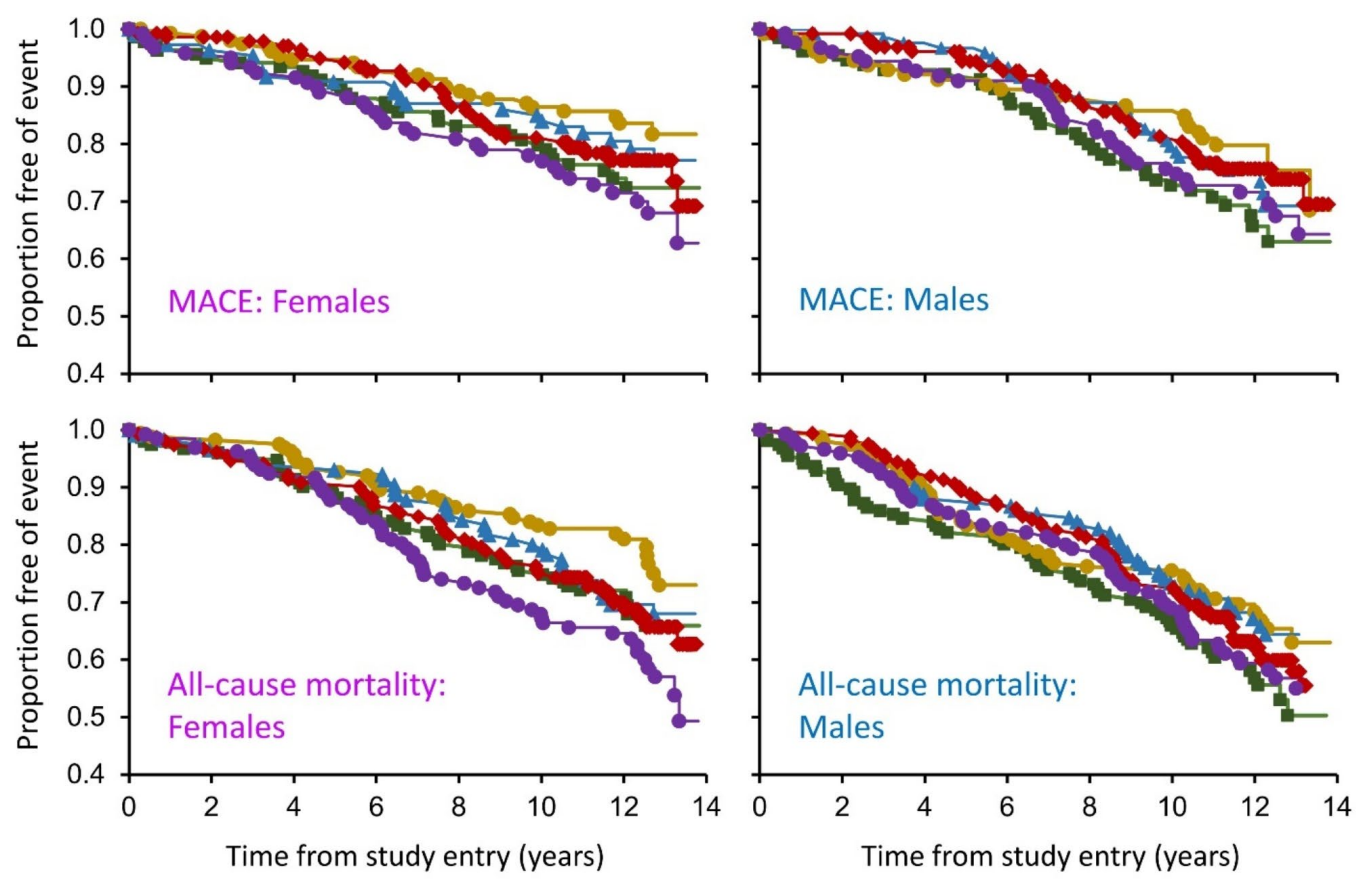
Kaplan-Meier plots of incident major adverse cardiovascular events (MACE; upper panels) and all-cause mortality (lower panels) for FDS2 participants in quintile 1 ν―ν, quintile 2 λ―λ, quintile 3 ▲―▲, quintile 4 ⬥―⬥ or quintile 5 λ―λ by sex (females left hand panels, males right hand panels)

The Kaplan-Meier curves for MACE by sex and serum HDL-cholesterol quintile are shown in Fig. 2. For females, quintiles 1 and 5 were significantly different to quintile 2 (*P* = 0.042 and *P* = 0.008, respectively). For males, there were no statistically significant differences in pairwise comparisons.

**Fig. 3:**
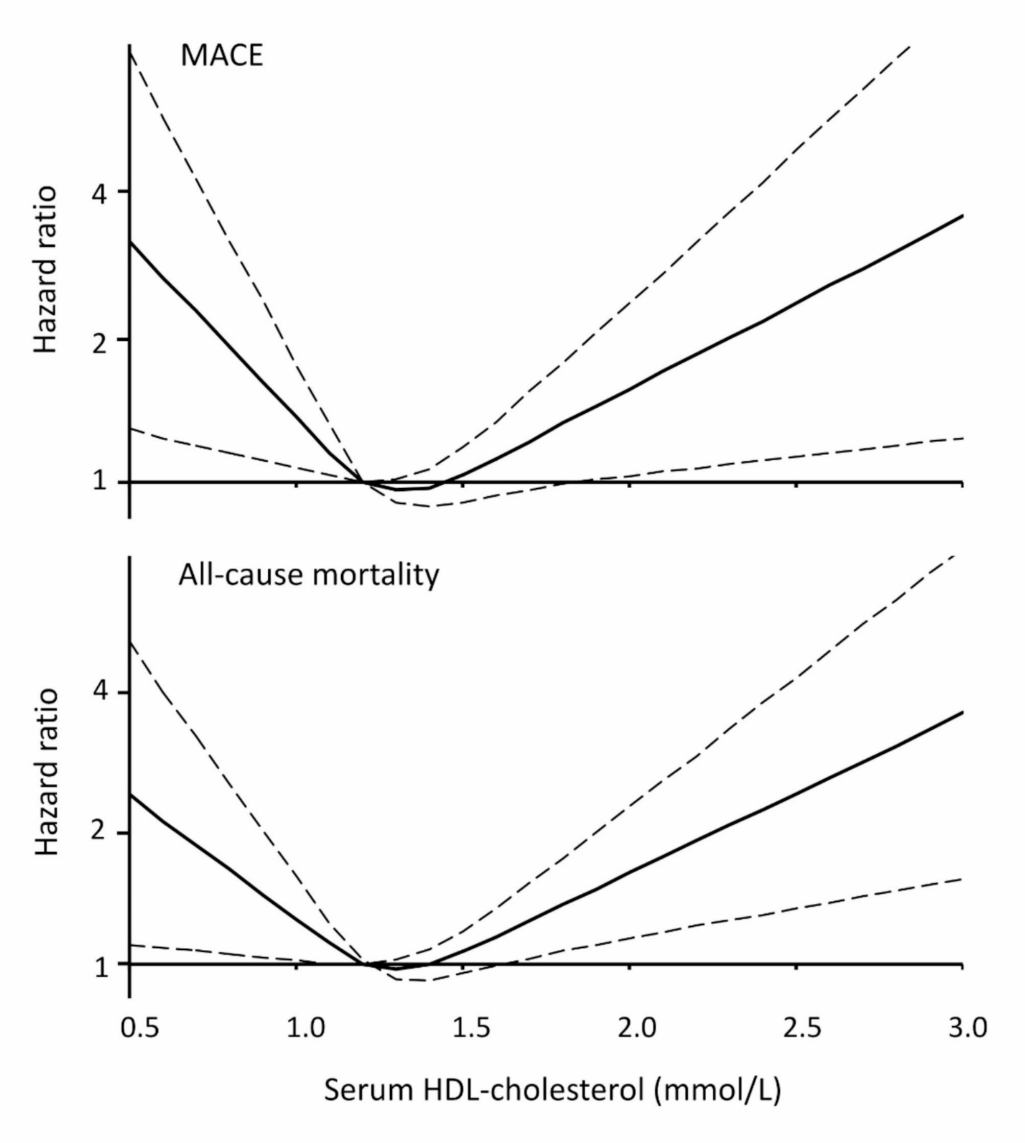
Relationship between serum HDL cholesterol and MACE (upper panel) and all-cause death (lower panel) in females using a 3 knot spline model with the lowest and highest values defining quintile 2 (1.04, 1.22 mmol/L (reference)) and the lowest end of quintile 5 (1.59 mmol/L)

The Cox regression model of independent predictors of incident MACE in females is summarised in Table 2. In addition to recognised demographic (increasing age and Aboriginal racial background), diabetes-specific (duration and glycemic control), other conventional (total serum cholesterol and PAD), and novel (serum NT-proBNP) risk factors, serum HDL-cholesterol quintiles showed a U-shaped relationship with this outcome. Compared with quintile 2 (1.04–1.22 mmol/L) both quintiles 1 (< 1.04 mmol/L) and 5 (> 1.59 mmol/L) showed a significantly increased risk in the final Cox model. This pattern was modestly attenuated after adjustment for the competing risk of death (Table 2). Figure 3 (upper panel) shows a cubic spline 3-knot model fitted to the data.

**Table 2:** Cox regression model of independent associates of three-point MACE in females with type 2 diabetes, excluding those with a prior history of MI or stroke, with female-specific quintiles of serum HDL-cholesterol added to the most parsimonious model excluding serum HDL-cholesterol

	Cox regression model csHR^a^ (95% CI)	*P*-value	Competing risk model sdHR^b^ (95% CI)	*P*-value
Age (increase of 10 years)	1.44 (1.21, 1.72)	< 0.001	1.31 (1.10, 1.55)	0.002
Aboriginal	2.54 (1.46, 4.43)	0.001	2.54 (1.47, 4.41)	0.001
Diabetes duration (increase of 5 years)	1.17 (1.06, 1.29)	0.002	1.14 (1.02, 1.26)	0.015
HbA_1c_ (increase of 1% or 11 mmol/mol)	1.20 (1.08, 1.33)	0.001	1.18 (1.07, 1.31)	0.002
Total serum cholesterol (increase of 1 mmol/L)	1.19 (1.07, 1.33)	0.001	1.15 (1.04, 1.27)	0.006
Ln(serum NT-proBNP (pg/mL))^c^	1.61 (1.40, 1.84)	< 0.001	1.44 (1.27, 1.65)	< 0.001
PAD	1.78 (1.26, 2.53)	0.001	1.58 (1.10, 2.28)	0.014
Serum HDL-cholesterol quintiles:				
1 (< 1.04 mmol/L)	1.90 (1.10, 3.28)	0.022	1.69 (0.99, 2.90)	0.056
2 (1.04–1.22 mmol/L)	1.00 (reference)		1.00 (reference)	
3 (1.23–1.38 mmol/L)	1.13 (0.63, 2.05)	0.679	1.21 (0.67, 2.17)	0.528
4 (1.39–1.59 mmol/L)	1.14 (0.66, 1.96)	0.649	1.10 (0.62, 1.96)	0.745
5 (> 1.59 mmol/L)	1.82 (1.07, 3.09)	0.027	1.73 (1.01, 2.97)	0.045

The competing risk of death from non-CVD causes was adjusted for in the competing risk model. There were 139 events in 653 participants in the final models

^a^Cause-specific hazard ratio; ^b^Sub-distribution hazard ratio; ^c^A 2.72-fold increase in x corresponds to an increase of 1 in ln(x)

The Cox regression model of independent predictors of incident MACE in males is summarised in Table 3. There were recognised demographic (increasing age), diabetes-specific (glycemic control) and other conventional (coronary heart disease, PAD and uACR) risk factors in the final Cox model, but serum HDL-cholesterol quintiles were not significant. This finding was also present after allowing for the competing risk of death from non-CVD causes (Table 3). Since no U-shaped relationship was apparent, no cubic spline model fitted the data.

**Table 3:** Cox regression model of independent associates of three-point MACE in males with type 2 diabetes, excluding those with a prior history of MI or stroke, with male-specific quintiles of serum HDL-cholesterol added to the most parsimonious model excluding serum HDL-cholesterol

	Cox model csHR^a^ (95% CI)	*P*-value	Competing risk model sdHR^b^ (95% CI)	*P*-value
Age (increase of 10 years)	1.59 (1.33, 1.90)	< 0.001	1.30 (1.11, 1.53)	0.001
HbA_1c_ (increase of 1% or 11 mmol/mol)	1.15 (1.02, 1.29)	0.018	1.14 (1.01, 1.29)	0.038
Coronary heart disease	1.47 (1.04, 2.08)	0.027	1.52 (1.07, 2.16)	0.020
PAD	1.58 (1.07, 2.33)	0.021	1.47 (1.005, 2.14)	0.047
Ln(urinary albumin: creatinine ratio (mg/mmol))^c^	1.19 (1.07, 1.34)	0.002	1.17 (1.03, 1.33)	0.013
Serum HDL-cholesterol quintiles:				
1 (< 0.90 mmol/L)	1.47 (0.89, 2.42)	0.130	1.28 (0.78, 2.12)	0.334
2 (0.90–1.02 mmol/L)	1.01 (0.59, 1.73)	0.972	0.93 (0.54, 1.59)	0.782
3 (1.03–1.14 mmol/L)	1.19 (0.71, 1.99)	0.501	1.05 (0.63, 1.75)	0.853
4 (1.15–1.31 mmol/L)	1.00 (reference)		1.00 (reference)	
5 (> 1.31 mmol/L)	1.07 (0.65, 1.76)	0.799	1.22 (0.74, 2.01)	0.432

The competing risk of death from non-CVD causes was adjusted for in the competing risk model. There were 156 events in 643 cases in the final models

^a^Cause-specific hazard ratio; ^b^Sub-distribution hazard ratio; ^c^A 2.72-fold increase in x corresponds to an increase of 1 in ln(x)

### All-cause mortality

Vital status was available in all participants at the end of follow-up (see Fig. 1). In the 713 females, 218 (30.6%) died during 10.6 ± 3.2 (range 0 to 13.8) years of follow-up. Compared to those who did not die in bivariable analyses (see Table 4), those who did were older, more likely Indigenous Australian and less likely Asian, were less likely to be married/in a *de facto* relationship, were less likely to have been educated beyond primary level, consumed less alcohol, were diagnosed younger and had longer duration diabetes, were more likely to be obese by ABSI and BMI, had higher systolic and lower diastolic blood pressures, had a higher heart rate, were more likely to be treated for hypertension, were more likely to have had prior CVD and were more likely to be taking aspirin, and had a higher likelihood of anaemia, thrombocytopenia, retinopathy, PAD, DSPN and nephropathy (lower eGFR and greater uACR). In relation to non-standard-of-care biomarkers and indices of liver dysfunction, those who died had higher serum NT-proBNP, serum AST/ALT, serum hyaluronic acid and serum alpha-2 macroglobulin, but a lower serum albumin. Their CCI was also significantly greater.

**Table 4:** Baseline characteristics by all-cause mortality to end-2021 by sex in participants from the Fremantle Diabetes Study Phase II with type 2 diabetes

	Female			Male		
	Alive	Deceased	*P-*value	Alive	Deceased	*P-*value
Number (%)	495 (69.4)	218 (30.6)		472 (61.9)	291 (38.1)	
Age (years)	62.5 ± 10.6	72.7 ± 11.5	< 0.001	61.8 ± 10.2	72.6 ± 9.4	< 0.001
Ethnic background (%):			0.042			0.018
Anglo-Celt	52.7	57.8		49.4	57.7	
Southern European	12.5	9.6		13.3	14.1	
Other European	6.3	6.0		9.1	5.5	
Asian	5.1	1.8		6.4	2.1	
Aboriginal	7.7	12.8		5.5	4.1	
Mixed/other	15.8	11.9		16.3	16.5	
Not fluent in English (%)	10.7	11.0	0.896	9.1	13.1	0.091
Currently married/*de facto* relationship (%)	60.0	34.4	< 0.001	75.8	65.3	0.002
Educated beyond primary level (%)	88.4	80.0	0.006	92.0	79.4	< 0.001
Smoking status (%)			0.063			0.045
Never	59.8	51.9		31.7	23.5	
Ex	32.3	35.6		56.6	64.7	
Current	7.9	12.5		11.7	11.8	
Alcohol consumption (standard drinks^a^/day)	0.1 [0-0.3]	0 [0-0.3]	0.004	0.8 [0.1–1.8]	0.3 [0-1.5]	0.006
Age at diabetes diagnosis (years)	54.2 ± 11.7	59.1 ± 14.2	< 0.001	53.0 ± 10.8	59.4 ± 11.9	< 0.001
Diabetes duration (years)	6.3 [2.0–14.0]	14.0 [5.0-19.9]	< 0.001	7.0 [2.0-14.8]	13.0 [5.0-18.1]	< 0.001
Diabetes treatment (%)			0.207			0.003
Diet-based	30.2	25.2		22.0	16.8	
Oral glucose lowering agents (OHAs)/non-insulin injectables	51.4	50.0		57.8	53.3	
Insulin only	4.7	5.5		4.0	9.6	
Insulin ± OHAs/non-insulin injectables	13.8	19.3		16.1	20.3	
Fasting serum glucose (mmol/L)	7.1 [6.1–8.6]	7.0 [6.0-8.9]	0.717	7.3 [6.3-9.0]	7.0 [6.0-8.4]	0.014
HbA_1c_ (%)	6.8 [6.2–7.6]	6.9 [6.3–7.9]	0.099	6.8 [6.3–7.8]	6.8 [6.2–7.5]	0.476
HbA_1c_ (mmol/mol)	51 [44–60]	53 [45–64]	0.024	51 [44–61]	53 [44–64]	0.166
BMI (kg/m^2^)	32.2 ± 6.5	30.7 ± 6.9	0.007	31.0 ± 5.5	30.2 ± 5.3	0.040
ABSI (m^11/6^ kg^− 2/3^)	0.079 ± 0.005	0.082 ± 0.006	< 0.001	0.082 ± 0.004	0.084 ± 0.004	< 0.001
Central obesity (by waist circumference^b^; %)	82.0	75.8	0.065	62.8	64.4	0.699
Systolic blood pressure (mmHg)	142 ± 21	148 ± 27	0.004	147 ± 19	150 ± 22	0.019
Diastolic blood pressure (mmHg)	78 ± 12	76 ± 14	0.049	84 ± 11	80 ± 12	< 0.001
Heart rate (beats/min)	71 ± 11	73 ± 14	0.010	67 ± 11	70 ± 14	0.005
Antihypertensive therapy (%)	69.6	79.6	0.006	71.0	81.0	0.002
Total serum cholesterol (mmol/L)	4.6 ± 1.1	4.6 ± 1.4	0.915	4.2 ± 1.0	4.0 ± 1.0	0.054
Serum non-HDL-cholesterol (mmol/L)	3.3 ± 1.1	3.2 ± 1.4	0.539	3.0 ± 1.0	2.9 ± 1.0	0.027
Serum HDL-cholesterol (mmol/L)	1.31 ± 0.32	1.39 ± 0.40	0.012	1.13 ± 0.29	1.15 ± 0.32	0.458
Serum HDL-cholesterol quintiles (%):			0.009			0.155
1	21.4	21.1		18.9	23.0	
2	25.9	16.1		21.8	16.8	
3	16.2	16.1		21.2	17.2	
4	21.0	22.0		20.6	21.6	
5	15.6	24.8		17.6	21.3	
Serum triglycerides (mmol/L)	1.6 (1.0-2.5)	1.5 (0.9–2.5)	0.630	1.5 (0.9–2.7)	1.5 (0.9–2.5)	0.335
Lipid-lowering therapy (%)	65.7	70.8	0.193	71.0	71.4	0.934
Statin use (%)	63.9	69.0	0.199	69.7	68.3	0.687
Aspirin therapy (%)	30.6	44.9	< 0.001	34.6	50.2	< 0.001
Cerebrovascular disease (%)	4.4	13.8	< 0.001	4.7	17.9	< 0.001
Coronary heart disease (%)	18.0	42.7	< 0.001	25.4	45.0	< 0.001
PAD (%)	22.1	39.6	< 0.001	12.7	27.7	< 0.001
DSPN (%)	24.1	44.9	< 0.001	40.4	58.7	< 0.001
eGFR category (%)			< 0.001			< 0.001
≥ 90 mL/min/1.73m^2^	47.4	20.3		46.6	21.0	
60–89 mL/min/1.73m^2^	43.5	45.2		45.5	47.4	
45–59 mL/min/1.73m^2^	5.7	17.1		6.0	13.7	
< 45 mL/min/1.73m^2^	3.5	17.5		1.9	17.9	
uACR (mg/mmol)	2.8 (0.8–9.1)	5.0 (1.3–19.2)	< 0.001	2.6 (0.7–9.6)	5.2 (1.1–23.8)	< 0.001
Any retinopathy (%)	27.6	45.5	< 0.001	39.7	44.0	0.252
Anaemia^c^ (%)	6.9	19.3	< 0.001	5.9	22.0	< 0.001
Platelets (x10^9^/L)	271 (214–344)	255 (189–343)	0.006	233 (182–297)	227 (163–316)	0.296
Serum NT-proBNP (pg/mL)	59 (20–174)	217 (53–889)	< 0.001	43 (11–160)	187 (39–892)	< 0.001
Serum hsCRP (mg/L)	3.1 (1.0-9.5)	2.9 (0.9–9.6)	0.510	1.9 (0.7–5.6)	2.4 (0.8–7.1)	0.018
Serum albumin (g/L)	44 ± 3	43 ± 4	0.003	45 ± 3	44 ± 3	< 0.001
Serum AST/ALT	1.1 (0.8–1.6)	1.4 (1.0-1.9)	< 0.001	1.0 (0.7–1.4)	1.3 (0.9–1.8)	< 0.001
Serum gamma-glutamyl transferase (U/L)	28 (15–53)	30 (14–67)	0.182	35 (18–68)	32 (14–74)	0.163
Serum bilirubin (µmol/L)	8.8 (5.9–13.3)	8.7 (5.6–13.4)	0.533	10.5 (7.1–15.6)	9.8 (6.6–14.7)	0.021
Serum apolipoprotein A1 (g/L)	1.56 ± 0.25	1.55 ± 0.30	0.800	1.41 ± 0.23	1.40 ± 0.26	0.473
Serum hyaluronic acid (ug/L)	44 (22–88)	74 (36–150)	< 0.001	49 (25–95)	77 (36–164)	< 0.001
Serum haptoglobin (g/L)	1.65 ± 0.56	1.65 ± 0.62	0.972	1.46 ± 0.57	1.55 ± 0.60	0.028
Serum alpha-2 macroglobulin (g/L)	1.92 (1.42–2.61)	2.51 (1.85–3.41)	< 0.001	1.96 (1.37–2.82)	2.36 (1.70–3.28)	< 0.001
CCI (%):			< 0.001			< 0.001
0	85.3	59.2		82.8	56.4	
1 or 2	10.7	26.1		15.0	25.4	
≥ 3	4.0	14.7		2.1	18.2	

^a^1 standard drink = 10 U ethanol; ^b^≥102 cm in males and ≥ 88 cm in females; ^c^haemoglobin ≤ 130 g/L males, ≤ 120 g/L females

Amongst the 763 males, 291 (38.1%) died during 10.1 ± 3.5 (range 0.2 to 13.9) years of follow-up. Compared to those who did not die, bivariable analyses (see Table 4) showed that those who did were older, were less likely to be Anglo-Celt, were less likely to be married/in a *de facto* relationship, were less likely to have been educated beyond primary level, were more likely to have never smoked, had a lower alcohol consumption, were diagnosed younger and had longer duration diabetes, were more likely to be obese by ABSI and BMI, had higher systolic and lower diastolic blood pressures, had a higher heart rate, were more likely to be treated for hypertension, were more likely to have had prior CVD and were more likely to be taking aspirin, and had a higher likelihood of anaemia, PAD, DSPN and nephropathy (lower eGFR and greater uACR). In relation to non-standard-of-care biomarkers and indices of liver dysfunction, those who died had higher serum hsCRP, serum NT-proBNP, serum AST/ALT, serum haptoglobin, serum hyaluronic acid and serum alpha-2 macroglobulin, but a lower serum, albumin. Their CCI was also significantly greater.

The Kaplan-Meier curves for mortality by sex and serum HDL-cholesterol quintile are shown in Fig. 2. For females, quintiles 1, 4 and 5 were significantly difference to quintile 2 (*P* = 0.036, *P* = 0.035 and *P* < 0.001, respectively). For males, quintile 1 was significantly different from quintile 2 (*P* = 0.047).

The Cox regression models of independent predictors of death in females are summarised in Table 5. In addition to recognised demographic (increasing age and Aboriginal racial background), conventional (ex-/current smoking, central obesity, increased heart rate, HbA_1c_, PAD, DSPN and an increased CCI), and novel (serum NT-proBNP and serum hyaluronic acid) risk factors, serum HDL-cholesterol quintiles showed a U-shaped relationship with this outcome. Compared with quintile 2 (1.04–1.22 mmol/L) both quintiles 1 (< 1.04 mmol/L) and 5 (> 1.59 mmol/L) showed a significantly increased risk in the final Cox model. Figure 3 (lower panel) shows a cubic spline 3-knot model fitted to the data.

**Table 5:** Cox models by sex of independent associates of all-cause mortality in type 2 diabetes with sex-specific quintiles of HDL-cholesterol added to the most parsimonious models excluding serum HDL-cholesterol

	Females		Males	
	Hazard ratio (95% CI)	*P*-value	Hazard ratio (95% CI)	*P-*value
Number of events/cases in final model (%)	210/692 (30.3)		286/745 (38.4)	
Age (increase of 10 years)	1.67 (1.39, 1.99)	< 0.001	2.31 (1.97, 2.71)	< 0.001
Aboriginal	1.78 (1.04, 3.05)	0.036		
Asian			0.34 (0.15, 0.79)	0.012
Currently married/*de facto*			0.74 (0.57, 0.96)	0.022
Ex-smoker	1.36 (1.002, 1.85)	0.048		
Current smoker	2.03 (1.23, 3.35)	0.006	1.65 (1.13, 2.42)	0.010
ABSI (increase of 0.001 m^11/6^ kg^− 2/3^)	1.06 (1.03, 1.08)	< 0.001		
Abdominal obesity (by waist circumference)	0.60 (0.41, 0.86)	0.006		
Heart rate (increase of 10 beats/min)	1.11 (0.997, 1.24)	0.057	1.51 (1.26, 1.82)	< 0.001
Time-varying heart rate			0.89 (0.81, 0.99)	0.027
Systolic blood pressure (increase of 10 mmHg)			0.80 (0.71, 0.91)	< 0.001
Time-varying systolic blood pressure			1.09 (1.02, 1.16)	0.011
HbA_1c_ (increase of 1% or 11 mmol/mol)	1.20 (1.09, 1.32)	< 0.001		
Insulin use			1.52 (1.16, 1.99)	0.002
Ln(serum NT-proBNP (pg/mL))^a^	1.50 (1.32, 1.70)	< 0.001	1.19 (1.07, 1.31)	0.001
PAD	1.41 (1.05, 1.89)	0.021	1.36 (1.03, 1.80)	0.031
DSPN	1.21 (1.04, 1.40)	0.012		
eGFR < 30 mL/min/1.73m^2^			2.00 (1.14, 3.49)	0.016
Ln(serum AST/ALT)^a^			1.36 (1.02, 1.82)	0.034
Serum albumin (g/L)			0.85 (0.79, 0.91)	< 0.001
Time-varying serum albumin			1.07 (1.03, 1.11)	0.001
Ln(serum hyaluronic acid (ug/L))^a^	1.40 (1.13, 1.72)	0.002	1.17 (0.98, 1.38)	0.085
CCI 1 or 2	2.26 (1.62, 3.14)	< 0.001		
CCI ≥ 3	2.33 (1.49, 3.64)	< 0.001	1.68 (1.18, 2.40)	0.004
Serum HDL-cholesterol quintiles:				
1	1.76 (1.10, 2.83)	0.019	1.53 (1.05, 2.21)	0.026
2	1.00 (reference)		1.06 (0.72, 1.56)	0.781
3	1.41 (0.86, 2.32)	0.170	1.11 (0.75, 1.63)	0.604
4	1.43 (0.90, 2.29)	0.131	1.00 (reference)	
5	2.02 (1.27, 3.23)	0.003	1.01 (0.70, 1.45)	0.956

^a^A 2.72-fold increase in x corresponds to an increase of 1 in ln(x)

The Cox regression models of independent predictors of death in males are summarised in Table 5. There were recognised demographic (increasing age and both inversely, being married/in a *de facto* relationship and Asian ethnicity), diabetes-specific (insulin use), conventional (current smoking, increased heart rate and lower systolic blood pressure which attenuated with time, PAD, serum AST/ALT, a lower serum albumin which attenuated with time, and a CCI ≥ 3), and novel (serum NT-proBNP and serum hyaluronic acid) risk factors. In addition, serum HDL-cholesterol quintile 1 (< 0.90 mmol/L) was associated with a significantly increased risk of death compared with reference quintile 4 (1.15–1.31 mmol/L). Since no U-shaped relationship was apparent, no cubic spline model fitted the data.

## Discussion

The present data show that, in a representative community-based cohort of people with type 2 diabetes, there was an independent U-shaped relationship between baseline quintiles of serum HDL-cholesterol and both MACE and all-cause death in females. For males, there was no association between serum HDL-cholesterol and MACE, but a serum HDL-cholesterol in the lowest quintile was a significant predictor of mortality after adjustment. These findings have implications for the sex-specific management of dyslipidaemia in the context of type 2 diabetes. Although there is no primary role for pharmacotherapy in raising a depressed serum HDL-cholesterol [30], there is an argument for using serum HDL-cholesterol concentrations to help guide risk assessment and preventive strategies differently for males and females [31].

In our females, the serum HDL-cholesterol nadir of risk for MACE and mortality was around 1.2 mmol/L (within the second quintile; see Fig. 3). There was a relatively steep increase in risk below, and a more gradual increment above, this point; an approximate 50% increase in risk of MACE/mortality was seen at a serum HDL-cholesterol < 0.9-1.0 mmol/L and > 1.8–1.9 mmol/L. For males, a serum HDL-cholesterol < 0.9 mmol/L was associated with an approximate 50% increased risk of death. It has been suggested that a serum HDL-cholesterol at these sorts of sex-specific thresholds could be seen as both a traditional risk factor and a risk enhancer [31], prompting consideration of additional diagnostic testing (such as lipoprotein(a) and/or coronary artery calcium scoring/CT angiography) and/or therapeutic intensification (high-potency statin and/or aspirin therapy) in primary prevention, and informing treatment intensification (such as combination or novel lipid-lowering therapy with proprotein convertase subtilisin/kexin type 9 inhibitors) in secondary prevention.

At present, this strategy awaits formal evaluation and is complicated by apparent inconsistencies in previously published data relating serum HDL-cholesterol to MACE and/or mortality in diabetes. Most [10–12] but not all [13] studies have found U-shaped relationships that were more prominent for females than males [10] or for males versus females [11, 12]. Interpretation of these differences is complicated by the fact that none of these studies included only people with type 2 diabetes, and there was a variable range and nature of potentially confounding variables used in analyses. The detailed FDS2 phenotypic data allowed clear differentiation of our participants with type 2 diabetes from those with other types, and the development of statistical models that included a full range of potentially confounding variables. Our data parallel those of the Pittsburgh Epidemiology of Diabetes Complications study in type 1 diabetes [10], but the discrepancies between the present and other studies [11–13] could reflect geo-epidemiological sex-specific differences in risk factors and management of CVD and other serious co-morbidities. In the case of the FDS2, we were able to adjust for key variables such as race/ethnicity, socioeconomic characteristics, diabetes-specific management and complications, use of cardiovascular risk-reducing therapies, and novel biomarkers including serum NT-proBNP and hsCRP.

Of these latter two analytes, serum NT-proBNP was a predictor of MACE and death in females, and of mortality in males, consistent with other studies in type 2 diabetes [32]. We also assessed the influence of variables associated with MAFLD and thus with potential confounding effects on serum HDL-cholesterol concentrations and the two outcomes of interest [25]. An inverse association between serum albumin concentrations and mortality has been found in MAFLD [33], but has also long been recognised in healthy individuals and patients with a variety of non-hepatic acute or chronic illnesses [34]. This relationship was evident in our male participants, as was a positive relationship between the AST: ALT ratio and death that has been found previously in type 2 diabetes [35]. Serum hyaluronic acid was a significant independent predictor of death in both sexes in the present study. We interpret this as a composite surrogate for the known positive associations between hyaluronic acid and mortality in chronic liver disease [36], chronic lung disease [37] and cancer [38].

The mechanisms underlying a U-shaped MACE/mortality relationship in females and a simpler inverse relationship with death at low serum HDL-concentrations in males are uncertain. Women have higher serum HDL-concentrations than in men in the general population, reflecting oestrogen-mediated generation of surface remnants from a greater rate of metabolism of VLDL-cholesterol to HDL-cholesterol, oestrogen-stimulated apolipoprotein A1 hepatic synthesis, and oestrogen-mediated reductions in hepatic lipase activity [39]. Diabetes reduces serum HDL-cholesterol but, as seen in the present study, women still have higher concentrations than men [40]. Type 2 diabetes impairs HDL-cholesterol function and this has adverse implications for its vascular protective effects [41]. This might help explain the association with low serum HDL-cholesterol concentrations and MACE in our women, but this was not observed in men in FDS2. High levels of HDL-cholesterol, with pro- rather than anti-inflammatory properties [42], may enhance vascular cholesterol deposition and consequent atherosclerosis [43], consistent with our MACE findings in women but not men. A U-shaped relationship has been found for all-cause mortality in a large Chinese study [7] which included evidence of this association for non-CVD causes of death, also revealed in other studies of infections [44], cancer [7] and renal disease [45]. Again, this was evident in our females but not males. These various observations infer a complex relationship between serum HDL-cholesterol, sex and MACE/mortality in type 2 diabetes.

On a practical level, the concept that the higher the serum HDL-cholesterol the better for cardiovascular and other outcomes has been challenged [46]. Perhaps because of the inconsistent findings in diabetes [10–13], the non-linearity of the relationship with MACE and mortality risk has not yet been addressed in recently published guidelines in diabetes [14]. Nevertheless, the present study suggests that risk assessment and preventive strategies should be prioritised in females with type 2 diabetes and a low or high serum HDL-cholesterol concentration, and in males with a low serum HDL-cholesterol.

The present study has limitations. Observational cohort studies can be affected by bias related to study recruitment. Our participants may have been relatively healthy, but their basic demographic and diabetes-related characteristics were similar to those of eligible people who were not recruited. Although we were able to ascertain vital status in all our participants during follow-up [16], it is possible that MACE events were incorrectly coded or missed. We did not include temporal changes in pharmacotherapy with potential implications for outcomes and follow-up serum HDL-cholesterol concentrations in our analyses. We did not have access to detailed data on diet and physical activity with potential influences on serum HDL-cholesterol concentrations but these have not been available in previous similar studies. The study strengths include the representative nature of the FDS2 cohort drawn from a catchment area typical of an urban Australian setting, the comprehensive nature of the baseline assessment, and the long-running validated WADLS through which the FDS2 has been linked with administrative data on all hospitalisations and deaths in the state of WA.

## Conclusions

The present study has shown that there are sex-specific relationships between serum HDL-cholesterol and both MACE and all-cause mortality in community-based people with type 2 diabetes. These findings should be confirmed in other cohorts but have potential implications for cardiovascular and mortality risk assessment and related clinical management strategies.

## Data Availability

Some outcome data supporting the findings of this study are available from the Western Australian Department of Health, but restrictions apply to the availability of these data, which were used under strict conditions of confidentiality for the current study, and so are not publicly available. Data are however available from the authors upon reasonable request and with permission of Western Australian Department of Health.

## Abbreviations

ABSI: A body shape index
AST/ALT: Serum aspartate/alanine transferase ratio
BMI: Body mass index
CCI: Charlson Comorbidity Index
CVD: Cardiovascular disease
DSPN: Distal symmetrical polyneuropathy
eGFR: Estimated glomerular filtration rate
FDS2: Fremantle Diabetes Study Phase II
hsCRP: High sensitivity C-reactive protein
HMDC: Hospital Morbidity Data Collection
IHD: Ischaemic heart disease
MACE: Major adverse cardiovascular events
MAFLD: Metabolic dysfunction-associated liver disease
MI: Myocardial infarction
NT-proBNP: N-terminal pro-brain natriuretic peptide
PAD: peripheral arterial disease
uACR: urinary albumin: creatinine ratio
WA: Western Australia
WADLS: WA Data Linkage System

## References

[1] Wilson PW, Abbott RD, Castelli WP. High density lipoprotein cholesterol and mortality. Framingham Heart Study Arterioscler. 1988;8(6):737–41. 10.1161/01.atv.8.6.737 3196218

[2] Barter PJ, Rye KA. High density lipoproteins and coronary heart disease. Atherosclerosis. 1996;121(1):1–12. 8678914 10.1016/0021-9150(95)05675-0

[3] Bowe B, Xie Y, Xian H, Balasubramanian S, Zayed MA, Al-Aly Z. High density lipoprotein cholesterol and the risk of all-cause mortality among U.S. Veterans. Clin J Am Soc Nephrol. 2016;11(10):1784–93. 27515591 10.2215/CJN.00730116 PMC5053782

[4] Hirata A, Okamura T, Sugiyama D, Kuwabara K, Kadota A, Fujiyoshi A, Miura K, Okuda N, Ohkubo T, Okayama A, et al. The relationship between very high levels of serum high-density lipoprotein cholesterol and cause-specific mortality in a 20-year follow-up study of Japanese General Population. J Atheroscler Thromb. 2016;23(7):800–9. 26923252 10.5551/jat.33449 PMC7399265

[5] Huang YQ, Liu XC, Lo K, Liu L, Yu YL, Chen CL, Huang JY, Feng YQ, Zhang B. The U shaped relationship between high-density lipoprotein cholesterol and all-cause or cause-specific mortality in adult population. Clin Interv Aging. 2020;15:1883–96. 33061337 10.2147/CIA.S271528 PMC7537851

[6] Ko DT, Alter DA, Guo H, Koh M, Lau G, Austin PC, Booth GL, Hogg W, Jackevicius CA, Lee DS, et al. High-density lipoprotein cholesterol and cause-specific mortality in individuals without previous Cardiovascular conditions: the CANHEART Study. J Am Coll Cardiol. 2016;68(19):2073–83. 27810046 10.1016/j.jacc.2016.08.038

[7] Lu J, Han G, Liu X, Chen B, Peng K, Shi Y, Zhang M, Yang Y, Cui J, Song L, et al. Association of high-density lipoprotein cholesterol with all-cause and cause-specific mortality in a Chinese population of 3.3 million adults: a prospective cohort study. Lancet Reg Health West Pac. 2024;42:100874. 38357392 10.1016/j.lanwpc.2023.100874 PMC10865023

[8] Madsen CM, Varbo A, Nordestgaard BG. Extreme high high-density lipoprotein cholesterol is paradoxically associated with high mortality in men and women: two prospective cohort studies. Eur Heart J. 2017;38(32):2478–86. 28419274 10.1093/eurheartj/ehx163

[9] Lui DTW, Tan KCB. High-density lipoprotein in diabetes: structural and functional relevance. J Diabetes Investig 2024. 10.1111/jdi.14172 PMC11215696 38416054

[10] Costacou T, Evans RW, Orchard TJ. High-density lipoprotein cholesterol in diabetes: Is higher always better? J Clin Lipidol. 2011;5(5):387–94. 21981840 10.1016/j.jacl.2011.06.011 PMC3190122

[11] Yan YQ, Chen J, Huang YQ. A non-linear association of high-density lipoprotein cholesterol with all-cause and cause-specific mortality in diabetic patients. Diabetes Metab Syndr Obes. 2021;14:2851–62. 34188508 10.2147/DMSO.S313006 PMC8235948

[12] Ishibashi T, Kaneko H, Matsuoka S, Suzuki Y, Ueno K, Ohno R, Okada A, Fujiu K, Michihata N, Jo T, et al. HDL cholesterol and clinical outcomes in diabetes mellitus. Eur J Prev Cardiol. 2023;30(8):646–53. 36738171 10.1093/eurjpc/zwad029

[13] Wu Z, Huang Z, Lichtenstein AH, Jin C, Chen S, Wu S, Gao X. Different associations between HDL cholesterol and cardiovascular diseases in people with diabetes mellitus and people without diabetes mellitus: a prospective community-based study. Am J Clin Nutr. 2021;114(3):907–13. 34019626 10.1093/ajcn/nqab163

[14] American Diabetes Association Professional Practice C. 10. Cardiovascular disease and risk management: standards of care in diabetes-2024. Diabetes Care. 2024;47(Suppl 1):S179–218. 38078592 10.2337/dc24-S010 PMC10725811

[15] Cooney MT, Dudina A, De Bacquer D, Fitzgerald A, Conroy R, Sans S, Menotti A, De Backer G, Jousilahti P, Keil U, et al. How much does HDL cholesterol add to risk estimation? A report from the SCORE investigators. Eur J Cardiovasc Prev Rehabil. 2009;16(3):304–14. 19609139 10.1097/HJR.0b013e3283213140

[16] Davis T, Bruce D, Davis W. Cohort profile: the Fremantle Diabetes Study. Int J Epidemiol. 2013;42(2):412–21. 22544845 10.1093/ije/dys065

[17] Socio-economic indexes for areas. http://www.abs.gov.au/websitedbs/censushome.nsf/home/seifa Accessed July 2024.

[18] Davis WA, Peters KE, Makepeace A, Griffiths S, Bundell C, Grant SFA, Ellard S, Hattersley AT, Paul Chubb SA, Bruce DG, et al. Prevalence of diabetes in Australia: insights from the Fremantle Diabetes Study Phase II. Intern Med J. 2018;48(7):803–9. 29512259 10.1111/imj.13792 PMC6037554

[19] Krakauer NY, Krakauer JC. Anthropometrics, Metabolic Syndrome, and Mortality Hazard. *J Obes* 2018, 2018:9241904. 10.1155/2018/9241904 PMC6079473 30123583

[20] Levey AS, Stevens LA, Schmid CH, Zhang YL, Castro AF 3rd, Feldman HI, Kusek JW, Eggers P, Van Lente F, Greene T, Coresh J. CKD-EPI (chronic kidney disease epidemiology collaboration). A new equation to estimate glomerular filtration rate. Ann Intern Med. 2009;150(9):604–12. 19414839 10.7326/0003-4819-150-9-200905050-00006 PMC2763564

[21] Davis WA, Hamilton E, Davis TME. Temporal trends in distal symmetric polyneuropathy in type 2 diabetes: the Fremantle Diabetes Study. J Clin Endocrinol Metab 2023. 10.1210/clinem/dgad646 PMC10876392 37930807

[22] Davis TM, Hunt K, McAullay D, Chubb SA, Sillars BA, Bruce DG, Davis WA. Continuing disparities in cardiovascular risk factors and complications between aboriginal and anglo-celt australians with type 2 diabetes: the Fremantle Diabetes Study. Diabetes Care. 2012;35(10):2005–11. 22815295 10.2337/dc12-0225 PMC3447856

[23] Succurro E, Marini MA, Fiorentino TV, Perticone M, Sciacqua A, Andreozzi F, Sesti G. Sex-specific differences in prevalence of nonalcoholic fatty liver disease in subjects with prediabetes and type 2 diabetes. Diabetes Res Clin Pr 2022, 190. 10.1016/j.diabres.2022.110027 35917992

[24] Tolman KG, Fonseca V, Dalpiaz A, Tan MH. Spectrum of liver disease in type 2 diabetes and management of patients with diabetes and liver disease. Diabetes Care. 2007;30(3):734–43. 17327353 10.2337/dc06-1539

[25] Davis TME. Diabetes and metabolic dysfunction-associated fatty liver disease. Metabolism 2021, 123. 10.1016/j.metabol.2021.154868 34400217

[26] Holman C, Bass A, Rouse I, Hobbs M. Population-based linkage of health records in Western Australia: development of a health services research linked database. Aust N Z J Public Health. 1999;23(5):453–9. 10575763 10.1111/j.1467-842x.1999.tb01297.x

[27] Charlson ME, Pompei P, Ales KL, MacKenzie CR. A new method of classifying prognostic comorbidity in longitudinal studies: development and validation. J Chronic Dis. 1987;40(5):373–83. 3558716 10.1016/0021-9681(87)90171-8

[28] Davis WA, Gregg EW, Davis TME. Temporal trends in Cardiovascular complications in people with or without type 2 diabetes: the Fremantle Diabetes Study. J Clin Endocrinol Metab 2020, 105(7). 10.1210/clinem/dgaa215 32352534

[29] Fine JP, Gray RJ. A proportional hazards model for the subdistribution of a competing risk. J Am Stat Assoc. 1999;94(446):496–509.

[30] Omari M, Alkhalil M. Atherosclerosis residual lipid risk-overview of existing and future pharmacotherapies. J Cardiovasc Dev Dis 2024, 11(4). 10.3390/jcdd11040126 PMC11050263 38667744

[31] Razavi AC, Mehta A, Jain V, Patel P, Liu C, Patel N, Eisenberg S, Vaccarino V, Isiadinso I, Sperling LS, et al. High-density lipoprotein cholesterol in atherosclerotic Cardiovascular Disease Risk Assessment: exploring and explaining the U-Shaped curve. Curr Cardiol Rep. 2023;25(12):1725–33. 37971636 10.1007/s11886-023-01987-3 PMC10898346

[32] Malachias MVB, Wijkman MO, Bertoluci MC. NT-proBNP as a predictor of death and cardiovascular events in patients with type 2 diabetes. Diabetol Metab Syndr. 2022;14(1):64. 35501909 10.1186/s13098-022-00837-6 PMC9063067

[33] Takahashi H, Kawanaka M, Fujii H, Iwaki M, Hayashi H, Toyoda H, Oeda S, Hyogo H, Morishita A, Munekage K et al. Association of Serum Albumin Levels and long-term prognosis in patients with biopsy-confirmed nonalcoholic fatty liver disease. Nutrients 2023, 15(9). 10.3390/nu15092014 PMC10180563 37432160

[34] Goldwasser P, Feldman J. Association of serum albumin and mortality risk. J Clin Epidemiol. 1997;50(6):693–703. 9250267 10.1016/s0895-4356(97)00015-2

[35] Zoppini G, Cacciatori V, Negri C, Stoico V, Lippi G, Targher G, Bonora E. The aspartate aminotransferase-to-alanine aminotransferase ratio predicts all-cause and cardiovascular mortality in patients with type 2 diabetes. Medicine. 2016;95(43):e4821. 27787357 10.1097/MD.0000000000004821 PMC5089086

[36] Plevris N, Sinha R, Hay AW, McDonald N, Plevris JN, Hayes PC. Index serum hyaluronic acid independently and accurately predicts mortality in patients with liver disease. Aliment Pharmacol Ther. 2018;48(4):423–30. 29971829 10.1111/apt.14897

[37] Papakonstantinou E, Bonovolias I, Roth M, Tamm M, Schumann D, Baty F, Louis R, Milenkovic B, Boersma W, Stieltjes B et al. Serum levels of hyaluronic acid are associated with COPD severity and predict survival. Eur Respir J 2019, 53(3). 10.1183/13993003.01183-2018 30705130

[38] Markowska A, Antoszczak M, Markowska J, Huczynski A. Role of Hyaluronic Acid in selected malignant neoplasms in Women. Biomedicines 2023, 11(2). 10.3390/biomedicines11020304 PMC9953106 36830841

[39] Knopp RH, Paramsothy P, Retzlaff BM, Fish B, Walden C, Dowdy A, Tsunehara C, Aikawa K, Cheung MC. Gender differences in lipoprotein metabolism and dietary response: basis in hormonal differences and implications for cardiovascular disease. Curr Atheroscler Rep. 2005;7(6):472–9. 16256006 10.1007/s11883-005-0065-6

[40] Walden CE, Knopp RH, Wahl PW, Beach KW, Strandness E Jr. Sex differences in the effect of diabetes mellitus on lipoprotein triglyceride and cholesterol concentrations. N Engl J Med. 1984;311(15):953–9. 6472421 10.1056/NEJM198410113111505

[41] Chapman MJ. HDL functionality in type 1 and type 2 diabetes: new insights. Curr Opin Endocrinol Diabetes Obes. 2022;29(2):112–23. 34980868 10.1097/MED.0000000000000705 PMC8915990

[42] Rosenson RS, Brewer HB Jr., Ansell BJ, Barter P, Chapman MJ, Heinecke JW, Kontush A, Tall AR, Webb NR. Dysfunctional HDL and atherosclerotic cardiovascular disease. Nat Rev Cardiol. 2016;13(1):48–60. 26323267 10.1038/nrcardio.2015.124 PMC6245940

[43] Furtado JD, Ruotolo G, Nicholls SJ, Dullea R, Carvajal-Gonzalez S, Sacks FM. Pharmacological inhibition of CETP (Cholesteryl Ester Transfer Protein) increases HDL (high-Density lipoprotein) that contains ApoC3 and other HDL subspecies Associated with higher risk of Coronary Heart Disease. Arterioscler Thromb Vasc Biol. 2022;42(2):227–37. 34937388 10.1161/ATVBAHA.121.317181 PMC8785774

[44] Madsen CM, Varbo A, Tybjaerg-Hansen A, Frikke-Schmidt R, Nordestgaard BG. U-shaped relationship of HDL and risk of infectious disease: two prospective population-based cohort studies. Eur Heart J. 2018;39(14):1181–90. 29228167 10.1093/eurheartj/ehx665

[45] Strazzella A, Ossoli A, Calabresi L. High-density lipoproteins and the kidney. Cells 2021, 10(4). 10.3390/cells10040764 PMC8065870 33807271

[46] Madsen CM, Nordestgaard BG. Is it time for new thinking about high-density lipoprotein? Arterioscler Thromb Vasc Biol. 2018;38(3):484–6. 29467220 10.1161/ATVBAHA.118.310727

